# Health-related quality of life of infants from ethnic minority groups: the Generation R Study

**DOI:** 10.1007/s11136-012-0184-9

**Published:** 2012-05-10

**Authors:** Ilse J. E. Flink, Tinneke M. J. Beirens, Caspar Looman, Jeanne M. Landgraf, Henning Tiemeier, Henriette A. Mol, Vincent W. V. Jaddoe, Albert Hofman, Johan P. Mackenbach, Hein Raat

**Affiliations:** 1 The Generation R Study Group, Erasmus MC - University Medical Center, Rotterdam, Rotterdam, The Netherlands; 2 Department of Public Health, Erasmus MC - University Medical Center, Rotterdam, P.O. Box 2040, 3000 CA Rotterdam, The Netherlands; 3 HealthActCHQ, Cambridge, MA USA; 4 Department of Child and Youth Psychiatry, Erasmus MC - University Medical Center, Rotterdam, Rotterdam, The Netherlands; 5 Department of Epidemiology, Erasmus MC - University Medical Center, Rotterdam, Rotterdam, The Netherlands; 6 Department of Pediatrics, Erasmus MC - University Medical Center, Rotterdam, Rotterdam, The Netherlands

**Keywords:** Infants, Health-related quality of life, Ethnicity, Migrants, Infant Toddler Quality of Life Questionnaire (ITQOL)

## Abstract

**Purpose:**

To assess whether the health-related quality of life of infants from ethnic minority groups differs from the health-related quality of life of native Dutch infants and to evaluate whether infant health and family characteristics explain the potential differences.

**Methods:**

We included 4,506 infants participating in the Generation R Study, a longitudinal birth cohort. When the child was 12 months, parents completed the Infant Toddler Quality of Life Questionnaire (ITQOL); ITQOL scale scores in each ethnic subgroup were compared with scores in the Dutch reference population. Influence of infant health and family characteristics on ITQOL scale scores were evaluated using multivariate regression models.

**Results:**

Infants from ethnic minority groups presented significantly lower ITQOL scale scores compared to the Dutch subgroup (e.g., Temperament and Moods scale: median score of Turkish subgroup, 70.8 (IQR, 15.3); median score of Dutch subgroup, 80.6 (IQR, 13.9; *P* < 0.001)). Infant health and family characteristics mediated an important part of the association between the ethnic minority status and infant health-related quality of life. However, these factors could not fully explain all the differences in the ITQOL scale scores.

**Conclusions:**

Parent-reported health-related quality of life is lower in infants from ethnic minority groups compared to native Dutch infants, which could partly be explained by infant health and by family characteristics.

## Introduction

Available empirical evidence suggests that infants and toddlers from ethnic minority groups may be at a disadvantage with regard to various health outcomes such as the prevalence of low birth weight [1, 2], behavioral problems [3] and respiratory symptoms [4, 5]. Children from ethnic minority groups more frequently grow up in adverse social circumstances like single-parent families and low socioeconomic position [6]. Based on these and other studies [7–9], it can be hypothesized that these negative health outcomes and adverse life circumstances also result in worse health-related quality of life, even in early life [10].

Some studies on the health-related quality of life of ethnic minority groups have been realized in older children. Pantzer et al. [11] showed that adolescents from ethnic minority groups in Spain (e.g., Latin American) reported a relatively lower health-related quality of life compared to their native counterparts. The presence of ethnic differences in health-related quality of life in early childhood has, to our knowledge, not been studied so far.

The first aim of this study was to assess whether the health-related quality of life of infants from ethnic minority groups differs from the health-related quality of life of native Dutch infants. Our hypothesis was that infants from ethnic minority groups would present worse health-related quality of life than native Dutch infants. In order to gain insight into the underlying mechanisms that may explain a potential difference, the second aim of this study was to evaluate to what extent infant health characteristics (birth weight, gestational age, presence of chronic conditions and wheezing) and family characteristics (marital status, educational level, family income and parental psychopathology) mediate the association between the ethnic minority status and infant health-related quality of life.

## Methods

### Design

This study was embedded in Generation R, a prospective population-based cohort from fetal life onwards [12]. Briefly, all pregnant women living in Rotterdam, the Netherlands, with an expected delivery date between April 2002 and January 2006 were invited to participate. The participation rate was estimated at 61 %. Written informed consent was obtained from all participants. The Medical Ethical Committee of the Erasmus University Medical Centre, Rotterdam, approved the study.

### Study population

**Fig. 1:**
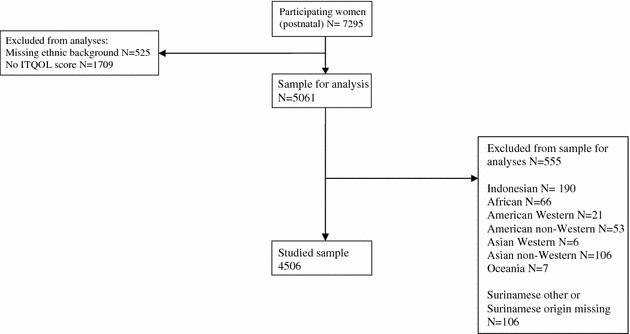
Flowchart of the study population

Full consent for the postnatal phase of the Generation R Study was obtained from 7,295 infants and their mothers. Women with missing data on their ethnic background (*N* = 525) were excluded. Infants for whom we did not have at least one ITQOL scale score were further excluded (*N* = 1,709). Due to small numbers of some ethnic subgroups or classification difficulties, 555 mothers were excluded (i.e., U.S.A. *N* = 74, Oceania *N* = 7, Asians *N* = 112, Indonesian *N* = 190, Africans *N* = 66 and Surinamese other *N* = 106). Figure 1 gives an overview of the study population.

### Measures

Data for this study were retrieved from medical records and collected by prenatal and postnatal questionnaires. On request, trained research assistants with varied ethnic backgrounds helped with completing the questionnaires.

#### Ethnic background

We classified the infants in the study population according to maternal ethnic background. A choice was made for maternal ethnic background because mothers play an important role in the lives of young children, and their cultural background and experiences of acculturation are most likely to influence their children [13]. Maternal ethnic background was determined by the country of birth of the mother and the mother’s parents [14]. If the mother or one of the mother’s parents was born abroad, this country of birth determined the national origin. If both parents were born abroad, the country of birth of the mother’s mother determined the ethnic background. Women with a Surinamese background were further classified as Surinamese Hindu or Surinamese Creole, based on self-classification. Subgroups of infants in the study were as follows: Dutch (*N* = 3,039), Other European (*N* = 415), Antillean (*N* = 97), Cape Verdean (*N* = 108), Surinamese Hindu (*N* = 93), Surinamese Creole (*N* = 89), Moroccan (*N* = 163) and Turkish (*N* = 302). Besides ethnic background, infant health-related quality of life may also be related to the generational status of the mother. As such, we established the generational status of non-Dutch participants. First generation included mothers who were born abroad; second generation included mothers who were born in the Netherlands.

#### Infant Toddler Quality of Life

Infant health-related quality of life was measured by the ITQOL, which was included in a questionnaire that was completed by the primary care giver at the age of 12 months of the child. The ITQOL covers both physical and psychosocial aspects and the impact of child health problems on family life [7, 15, 16]. The full-length research version of the ITQOL consists of 103 items (10 multi-item scales and 2 single-item scales; see Table 1) that generally refer to the situation during the past 4 weeks. Per scale, the items that have 4, 5 or 6 response options were summed up with equal weight per item (some recoded and/or recalibrated) and transformed into a 0 (worst possible score) to 100 (best possible score) scale [7, 15, 16]. The ITQOL General behavior and Getting along scales, and the single item Change in Health were not included in the study since they are relevant to children aged older than 12 months [7, 15, 16]. The ITQOL questionnaire was available in Dutch, English and Turkish. The great majority (95.3 %) filled in the Dutch version, 1.3 % filled in the English version and 3.4 % filled in the Turkish version. Good reliability and validity have been reported for the Dutch and English versions of the ITQOL [7, 15]. In our sample, internal consistencies for the ITQOL scales of the Dutch version ranged from α = 0.97 for Physical Functioning to α = 0.75 for General Health. Internal consistencies for the ITQOL scales of the English and Turkish versions were similar to the Dutch version.

**Table 1:** ITQOL-scales, number of items per scale and score interpretation

Scale	Number of items	Description low score	Description high score
Physical functioning	10	Child is considerably limited in performing physical activities such as eating, sleeping, grasping and playing due to health problems	Child performs all types of physical activities such as eating, sleeping, grasping and playing without limitations due to health problems
Growth and development	10	Parent is very dissatisfied with development (physical growth, motor, language, cognitive), habits (eating, feeding, sleeping) and overall temperament	Parent is very satisfied with development (physical growth, motor, language, cognitive), habits (eating, feeding, sleeping) and overall temperament
Bodily pain	3	Child has extremely severe, frequent and limiting bodily pain/discomfort	Child has no pain or limitations due to pain/discomfort
Temperament and moods	18	Child very often has certain moods and temperaments, such as sleeping/eating difficulties, crankiness, fussiness unresponsiveness and lack of playfulness and alertness	Child never has certain moods and temperaments, such as sleeping/eating difficulties, crankiness, fussiness unresponsiveness and lack of playfulness and alertness
General behavior^a^	13	Parent believes child’s behavior is poor and likely to get worse	Parent believes child’s behavior is excellent and will continue to be so
Getting along^a^		Child very often exhibits behavior problems, such as not following directions, hitting and biting others, throwing tantrums, and being easily distracted, while positive behaviors, such as ability to cooperate, appear sorry and adjustment to new situations are seldom shown	Child never exhibits behavior problems, such as not following directions, hitting and biting others, throwing tantrums, and being easily distracted, while positive behaviors, such as ability to cooperate, appear sorry and adjustment to new situations are frequently shown
General health perceptions	12	Parent believes child’s health is poor and likely to get worse	Parent believes child’s health is excellent and will continue as such
Parental impact: emotional	7	Parent experiences a great deal of emotional worry/concern as a result of child’s physical and/or psychosocial health and/or growth and development	Parent does not experience feelings of emotional worry/concern as a result of child’s physical and/or psychosocial health and/or growth and development
Parental impact: time	7	Parent experiences a lot of limitations in time available for personal needs due to child’s physical and/or psychosocial health and/or growth and development	Parent does not experience limitations in time available for personal needs due to child’s physical and/or psychosocial health and/or growth and development
Family activities	6	The child’s health and/or growth and development very often limits and interrupts family activities or is a source of family tension	The child’s health and/or growth and development never limits and interrupts family activities or is a source of family tension
Family cohesion	1	Family’s ability to get along is rated as “poor”	Family’s ability to get along is rated as “excellent”
Change in Health^a^	1	Child’s health is much worse now than 1 year ago	Child’s health is much better now than 1 year ago

Reproduced with permission from the principal author Landgraf [52] and ^©^2010 HealthActCHQ. All rights reserved

^a^Only applicable to children aged older than 1 year

#### Potential confounders and mediators

The following variables were considered to influence the association between maternal ethnic background and health-related quality of life in infants 12 months of age. These were selected based on current literature on determinants of health-related quality of life in children [8–10, 17, 18]. Maternal age, parity (the number of live births the mother delivered before the participating child), the child’s gender and age of the child during the questionnaire were treated as confounders.

Infant health characteristics that were considered to be potential mediators were child’s birth weight (≤2,500 or >2,500 g) [1], gestational age at birth (≤36 weeks or >36 weeks), presence of chronic conditions the past 6 months (<1 or ≥1) [19] and presence of episodes of wheezing the past year [20].

Family characteristics that were considered to be potential mediators were as follows: marital status of the mother (married/cohabiting or no partner); highest attained educational level of the mother—low (i.e., primary school, lower vocational training, intermediate general school or 3 years general secondary school); medium (i.e. >3 years general secondary school, intermediate vocational training or 1st year higher vocational training); high (i.e. higher vocational training, Bachelor’s degree, higher academic education or PhD) [8]—family income (low, i.e., <1,200 €, which is below social security level; 1,200–2,000 €; >2,000 €, which is more than modal income) [9, 17]; and level of prenatal maternal psychopathology, measured during pregnancy by the Global Severity Index of the Brief Symptom Inventory (BSI), a validated self-report questionnaire with 53 items [21, 22]. The internal consistency of the Global Severity Index, the overall score of the BSI, in this sample was α = 0.96.

### Statistical analyses

Statistical analyses were performed using SPSS for windows, version 18 (SPSS Inc., Chicago, IL). Frequency tables and cross-tabulations were used to explore characteristics of the study population, stratified by maternal ethnic background (Table 2). Median (IQR) scores on the ITQOL scales were calculated for each ethnic subgroup. Mann–Whitney *U* tests were performed to evaluate the statistical differences in ITQOL scores between ethnic subgroups and the Dutch reference subgroup, given a non-normal distribution of some of the ITQOL scales (Tables 3, 4). To check whether generational status of the non-Dutch groups was of importance, we also compared median (IQR) scores of children whose mothers were first-generation immigrants and whose mothers were second-generation immigrants to the native Dutch group. To adjust for multiple testing, the significance level was Bonferroni corrected (α/k) [23].

**Table 2:** Child and family characteristics

	*N*	Dutch	Other European	Antillean	Cape Verdean	Surinamese Creole	Surinamese Hindu	Moroccan	Turkish	*P* value
		*N* = 3,239	*N* = 415	*N* = 97	*N* = 108	*N* = 89	*N* = 93	*N* = 163	*N* = 302	
Infant characteristics										
Gender (% boys)	4,506	50.4	46.3	36.1	43.5	52.8	46.2	44.8	50.7	0.06
Age (months)	4,493	12.6 (1.8)	12.7 (2.3)	13.1 (2.7)	12.7 (1.5)	13.2 (2.5)	12.9 (1.7)	12.8 (1.4)	12.8 (2.1)	0.24
Birth weight (% ≤2,500)	4,504	4.7	5.1	7.2	7.4	6.7	10.8	2.5	3.6	0.06
Gestational age at birth (% ≤36 weeks)	4,505	3.3	3.2	3.2	1	2.3	1.1	1.2	3	0.62
Number of chronic conditions past 6 months (% ≥1)	4,506	78.1	73.7	74.2	76.9	82	76.3	79.8	88.4	0.001
Wheezing past 12 months (% yes)	4,506	29.4	27.5	26.8	32.4	27	28	23.9	34.1	0.39
Family characteristics										
Age of mother at intake (years)	4,506	32.0 (4.0)	31.7 (4.3)	27.6 (5.3)	28.6 (5.8)	30.2 (5.7)	28.4 (4.7)	28.9 (5.3)	27.7 (5.1)	<0.001
Educational level of mother	4,442									
High (%)		65.3	67.4	23.2	15.2	24.1	18.5	16.3	15.6	<0.001
Medium (%)		32.9	29.3	67.4	66.7	65.5	70	58.2	54.2	
Low (%)		1.8	3.3	9.5	18.1	10.3	10.9	25.5	30.2	
Family net income (Euros)										
>2,000	3,872	85.3	77.5	25	20.2	39.7	21.6	18	39.5	<0.001
1,200–2,000		11.1	16.3	25	25	28.8	28.9	37.8	38	
<1,200		3.5	6.2	50	54.8	31.5	31.6	44.1	40.4	
Marital status mother (% single)	4,416	5.2	5.1	42.3	46.6	41.6	18.3	3.1	5.1	<0.001
Prenatal maternal psychopathology^a,b^	3,724	0.12	0.15	0.26	0.36	0.19	0.28	0.27	0.38	<0.001
		(0.06, 0.23)	(0.06, 0.31)	(0.12, 0.48)	(0.14, 0.79)	(0.10, 0.35)	(0.08, 0.56)	(0.12, 0.66)	(0.17, 0.75)	
Parity (% nulli)	4,425	60.7	62.6	63.5	51.9	57.3	59.8	40.7	51	<0.001
Respondent (% mother)	4,269	85.9	82.4	83.9	87.5	92.8	84.3	82.2	87.8	0.18

Values are percentages and means (SD) (except for maternal psychopathology). *P* values are for chi-square test for categorical variables, analysis of variance (ANOVA) for continuous normally distributed variables and the Kruskal–Wallis test for continuous non-normally distributed variables

^a^Global severity index of the brief symptom inventory

^b^Median (IQR)

**Table 3:** Median (IQR) scores on ITQOL scales stratified by maternal ethnic background

ITQOL scale	Dutch (reference)	Other European	Antillean	Cape Verdean	Surinamese Creole	Surinamese Hindu	Moroccan	Turkish
	*N* = 3,239	*N* = 415	*N* = 97	*N* = 108	*N* = 89	*N* = 93	*N* = 163	*N* = 302
	Median (IQR)	Median (IQR)	Median (IQR)	Median (IQR)	Median (IQR)	Median (IQR)	Median (IQR)	Median (IQR)
PF	100 (3.7)	100 (3.3)	100 (13.0)	100 (11.1)	100 (3.3)	100 (18.5)*	100 (20.0)*	100 (16.7)*
GD	92.5 (15.0)	92.5 (15.0)	90.0 (20.0)	90.8 (17.5)	91.3 (20.0)	82.0 (22.5)*	85.0 (22.5)*	87.5 (22.5)*
BP	66.7 (13.3)	66.7 (13.3)	66.7 (13.3)	66.7 (13.3)	66.7 (20.0)*	60.0 (26.7)	66.7 (16.7)	60.0 (20.0)
TM	80.6 (13.9)	79.2 (15.3)*	77.8 (15.3)	76.4 (16.6)*	79.2 (12.5)	71.5 (16.6)*	70.8 (18.1)*	70.8 (15.3)*
GH	85.4 (15.8)	83.3 (16.8)	80.6 (16.6)*	83.3 (14.5)	84.8 (16.3)	81.3 (19.4)	77.9 (22.5)*	79.6 (20.8)*
PE	96.4 (7.1)	96.4 (10.7)	95.8 (10.7)*	96.4 (10.7)	96.4 (10.7)	92.9 (14.3)*	96.4 (10.7)	92.9 (16.7)*
PT	95.2 (9.5)	95.2 (14.3)	95.2 (14.3)	95.2 (14.3)	100 (9.5)*	95.2 (19.0)	95.2 (23.8)*	90.5 (19.0)*
FA	91.7 (16.7)	91.7 (25.0)	91.7 (25.0)	88.8 (25.0)	91.7 (20.8)	83.3 (25.0)*	87.5 (29.2)*	83.3 (29.2)*
FC	85.0 (15.0)	85.0 (15.0)	85.0 (27.5)*	85.0 (27.5)*	85.0 (42.5)*	85.0 (42.5)*	85.0 (42.5)*	85.0 (42.5)*

*P* values are based on the Mann–Whitney *U* test for differences in ITQOL scale scores between the subgroups and the reference group. α level is Bonferroni corrected

*PF* physical functioning, *GD* growth and development, *BP* bodily pain, *TM* temperament and moods, *GH* general health perceptions, *PE* parental impact emotional, *PT* parental impact time, *FA* family activities, *FC* family cohesion

* *P* value <0.007 indicates a statistically significant difference from Dutch subgroup

**Table 4:** Relative differences in infant health-related quality of life by maternal ethnic background after adjustment for confounders and mediators

ITQOL Scale	Other European	Antillean	Cape Verdean	Surinamese Creole	Surinamese Hindu	Moroccan	Turkish
	*N* = 389	*N* = 91	*N* = 96	*N* = 86	*N* = 91	*N* = 145	*N* = 281
PF							
Model 1	−1.0 (−7.2; 3.9)	−7.2 (−16.1; 1.2)	−**32.1 (**−**52.4;** −**10.7)**	−7.0 (−22.0; 5.3)	−**28.3 (**−**46.2;** −**9.4)**	−**21.7 (**−**35.4;** −**8.8)**	−**21.6 (**−**31.4;** −**12.3)**
Model 2	−1.2 (−7.4; 3.7)	−7.5 (−16.3; 0.8)	−**32.1 (**−**52.4;** −**10.8)**	−6.6 (−10.8; 5.4)	−**28.0 (**−**46.3;** −**9.4)**	−**21.6 (**−**35.3;** −**8.7)**	−**21.2 (**−**31.2;** −**11.8)**
Model 3	−1.8 (−2.5; 5.2)	−4.3 (−14.7; 4.8)***	−**30.4 (**−**51.4;** −**7.6)**	−5.8 (−22.9; 9.2)**	−18.4 (−37.9; 1.4)***	−6.3 (−19.7; 6.6)***	−11.3 (−21.8; 0.4)***
GD							
Model 1	0.2 (−1.2; 1.5)	−2.3 (−5.3; 0.6)	0.6 (−2.1; 3.5)	−1.0 (−4.3; 2.2)	−**3.5 (**−**11.7;** −**0.6)**	−**7.9 (**−**16.1;** −**2.6)**	−**5.7 (**−**11.7;** −**2.0)**
Model 2	0.0 (−1.3; 1.4)	−2.3 (−5.4; 0.6)	0.6 (−2.2; 3.5)	−0.9 (−4.3; 2.2)	−**3.4 (**−**6.3;** −**0.6)**	−**8.0 (**−**16.2;** −**2.6)**	−**5.5 (**−**11.4;** −**1.6)**
Model 3	0.4 (−0.9; 1.8)**	−1.4 (−4.6; 2.1)	3.0 (−0.3; 6.8)*	−0.3 (−4.4; 3.7)	−2.0 (−4.8; 1.2)	−**7.5 (**−**17.0;** −1.1)*	−4.1 (−10.8; 0.2)
BP							
Model 1	1.2 (−1.8; 3.7)	−2.7 (−9.3; 3.9)	**4.7 (0.8; 8.9)**	**7.1 (1.8; 12.2)**	0.8 (−5.2; 6.5)	1.4 (−2.5; 5.6)	−**5.9 (**−**11.8;** −**0.2)**
Model 2	0.6 (−2.2; 3.2)*	−3.0 (−9.7; 3.3)	**5.2 (1.3; 9.4)**	**7.2 (2.2; 12.2)**	0.8 (−5.1; 6.5)	1.4 (−2.5; 5.6)	−4.9 (−11.0; 0.9)***
Model 3	1.6 (−1.4; 4.2)*	−4.1 (−11.5; 3.2)	**7.6 (2.9; 13.0)**	**8.2 (2.0; 14.3)**	1.3 (−5.4; 7.8)	0.9 (−3.7; 5.8)	−4.4 (−10.5; 1.5)
TM							
Model 1	−1.3 (−2.8; 0.3)	−3.4 (−7.4; 0.4)	−2.1 (−5.1; 1.2)	−1.1 (−4.3; 1.9)	−**9.0 (**−**13.0;** −**5.4)**	−**10.2 (**−**13.2;** −**7.0)**	−**10.7 (**−**12.9;** −**8.2)**
Model 2	−1.6 (−3.1; 0.1)*	−3.5 (−7.5; 0.6)	−1.9 (−4.7; 1.4)	−1.0 (−4.4; 2.1)	−**7.2 (**−**12.9;** −**5.4)**	−**10.2 (**−**13.2;** −**7.1)**	−**10.2 (**−**12.6;** −7.7)***
Model 3	−**0.9 (**−**2.3;** −0.8)**	−3.2 (−8.0; 1.2)	0.5 (−3.0; 4.6)**	1.0 (−4.9; 2.6)	−**7.2 (**−**11.9;** −3.1)*	−**7.9 (**−**11.4;** −4.1)*	−**7.1 (**−**9.9;** −4.3)***
GH							
Model 1	−1.1 (−3.2; 1.0)	−**5.9 (**−**12.4;** −**0.5)**	−2.4 (−6.7; 1.5)	1.0 (−3.0; 4.5)	−2.3 (−6.6; 1.7)	−**8.6 (**−**12.6;** −**4.9)**	−**4.6 (**−**7.4;** −**2.1)**
Model 2	−1.8 (−3.7; 0.2)	−**5.8 (**−**12.2;** −**0.6)**	−1.7 (−6.0; 2.2)	0.3 (−3.0; 4.2)	−2.3 (−6.2; 1.7)	−**8.8 (**−**12.7;** −**5.2)**	−**3.6 (**−**6.3;** −1.1)*
Model 3	−1.0 (−3.1; 1.0)***	−**6.2 (**−**13.5;** −**0.3)**	0.3 (−4.6; 3.8)	0.3 (−4.0; 4.3)	−1.1 (−6.1; 3.5)	−**7.9 (**−**11.7;** −2.6)**	−1.7 (−4.5; 1.0)*
PE							
Model 1	−0.9 (−2.2: 0.2)	−**4.9 (**−**9.1;** −**1.4)**	−**6.4 (**−**12.3;** −**1.4)**	−2.1 (−6.4; 1.2)	−**9.8 (**−**14.9;** −**4.7)**	−**3.7 (**−**7.4;** −**0.7)**	−**7.2 (**−**9.9;** −**4.4)**
Model 2	−**1.2 (**−**2.5;** −0.1)*	−**5.0 (9.3;** −**1.4)**	−**6.3 (**−**12.2;** −**1.3)**	−2.2 (−6.4; 1.0)	−**9.9 (**−**14.9;** −**4.9)**	−**3.7 (**−**7.5;** −**0.8)**	−**6.8 (**−**9.5;** −4.2)*
Model 3	−0.9 (−2.4; 0.3)	−4.6 (−9.9; 0.1)	−4.3 (−11.3; 1.8)	−2.0 (−6.8; 1.8)	−**7.6 (**−**12.8;** −**3.1)**	−2.1 (−6.3; 1.2)	−**3.8 (**−**6.3;** −1.3)***
PT							
Model 1	0.6 (−3.4; 4.2)	−9.2 (−22.9; 1.3)	−11.1 (−28.1; 4.1)	3.4 (−0.6; 7.1)	−8.6 (−22.0; 1.6)	−5.5 (−11.3; 0.2)	−**13.7 (**−**21.8;** −**6.8)**
Model 2	0.5 (−3.4; 4.1)	−9.4 (−23.2; 1.1)	−11.4 (−28.6; 3.8)	3.5 (−0.5; 7.3)	−8.6 (−11.0; 1.8)	−5.6 (−11.3; 0.2)	−**13.5 (**−**21.7;** −**6.5)**
Model 3	1.2 (−2.6; 4.9)	−12.3 (−27.2; 0.0)	−11.0 (−30.7; 7.8)	4.7 (−0.6; 10.0)	−8.3 (−23.5; 3.3)	−7.6 (−15.4; 0.3)	−**12.3 (**−**22.5;** −**2.2)**
FA							
Model 1	−2.7 (−6.5; 0.4)	−2.6 (−7.2; 2.3)	−5.6 (−11.8; 0.4)	1.2 (−6.3; 3.1)	−**8.3 (**−**14.2;** −**2.5)**	−**9.3 (**−**15.0;** −**3.5)**	−**9.1 (**−**12.4;** −**5.5)**
Model 2	−3.1 (6.8; 0.0)	−2.7 (−7.5; 2.0)	−5.5 (−11.7; 0.2)	−1.2 (−6.1; 3.1)	−**8.0 (**−**13.8;** −**2.3)**	−**9.3 (**−**15.2;** −**3.5)**	−**8.5 (**−**11.9;** −4.8)*
Model 3	−1.8 (−5.4; 1.2)**	−2.8 (−8.1; 2.8)***	0.7 (−6.7; 8.5)***	0.2 (−5.9; 5.0)	−5.6 (−12.2; 0.7)	−**8.0 (**−**14.7;** −1.1)***	−4.2 (−8.6; 0.3)***
FC							
Model 1	−**5.6 (**−**10.6;** −**1.5)**	−**12.2 (**−**18.2;** −**6.1)**	−**16.1 (**−**21.8;** −**9.6)**	−**10.5 (**−**16.5;** −**4.9)**	−**10.4 (**−**16.4;** −**4.7)**	−**7.7 (**−**12.2;** −**3.6)**	−**11.4 (**−**17.4;** −**6.3)**
Model 2	−**5.7 (**−**10.6;** −**1.6)**	−**12.4 (**−**18.2;** −**6.0)**	−**15.9 (**−**22.0;** −**9.9)**	−**10.5 (**−**16.3;** −**4.8)**	−**11.1 (**−**16.4;** −**4.7)**	−**7.9 (**−**12.3;** −**3.7)**	−**11.4 (**−**17.2.;** −**6.3)**
Model 3	−4.3 (−4.7; 0.4)**	−**8.9 (**−**15.0;** −1.0)**	−**8.4 (**−**14.0;** −1.2)***	−6.8 (−13.2; 0.6)**	−6.6 (−9.4; 2.5)***	−2.9 (−7.7; 4.2)**	−6.1 (−5.5; 3.0)***

Values are effect sizes representing relative differences (%) in health-related quality of life scores compared to the Dutch reference group and 95 % confidence intervals estimated by bootstrap analyses. Relative differences calculated as follows: ((Exp(β) − 1) × 100). Bold values indicate a significant difference compared to Dutch group

Model 1 is adjusted for confounders: gender, age of child, age of mother and parity. Model 2 is adjusted for confounders and infant health characteristics: birth weight, gestational age, presence of chronic conditions and wheezing. Model 3 is adjusted for confounders, infant health characteristics and family characteristics: maternal educational level, marital status, family income and maternal psychopathology

*PF* physical functioning, *GD* growth and development, *BP* bodily pain, *TM* temperament and moods, *GH* general health perceptions, *PE* parental impact emotional, *PT* parental impact time, *FA* family activities, *FC* family cohesion

* *P* value <0.05; ** *P* value at <0.01; *** *P* value <0.001 indicate whether the strength of the association between maternal ethnic background and infant health-related quality of life changed significantly after adding a set of variables to the model as derived from the bootstrap analysis

Given the non-normal distribution of some of the ITQOL scales, we conducted log transformations (log(y) + 1) of all ITQOL scales. Hereafter, multivariate linear regression analyses were performed to test the association between maternal ethnic background and infant HRQOL. Model 1 was the association between maternal ethnic background and infant HRQOL adjusted for potential confounders. Subsequently, in model 2 we added infant health characteristics to model 1. In model 3 we added family characteristics to model 2. For each covariate, an interaction term with maternal ethnic background was tested for significance. Maternal educational level, marital status and family income interacted significantly (*P* < 0.05) with maternal ethnic background in close to half of the ITQOL scales. When stratifying the analyses by these variables, the associations were in the same direction and we therefore do not show stratified analyses.

The bootstrap procedure was used to estimate *P* values, standard errors (SE) and confidence intervals (CI). The bootstrap was also used to examine whether the strength in association between ethnic background and infant HRQOL changed significantly after adding a set of variables to the model. The bootstrap is a data-based simulation method for analyzing data including hypothesis testing (*P* values), standard errors (SE) and confidence interval (CI) estimation that does not rely on the assumptions of normality [24, 25]. It repeatedly draws random samples from the original data, with replacement [26]. The bootstrap procedure was conducted in R version 2.7.1 [27].

For each model, effect sizes representing the relative differences (%) in health-related quality of life scores of non-Dutch groups compared to the Dutch reference group were calculated using the following formula ((exp(β) − 1) × 100). The relative differences and corresponding 95 % confidence intervals (CIs) are presented in Table 4.

### Non-response analyses

Within the Dutch subgroup, mothers with no outcome for any of the ITQOL scales at 12 months (*n* = 1,272) were compared with those mothers for whom we had at least one ITQOL scale outcome (*n* = 3,239). Data on the ITQOL were more often missing in mothers who were lower educated (χ^2^ = 190.1; *P* < 0.001), single parents (χ^2^ = 73.0; *P* < 0.001) and younger than 25 years when included in the study (χ^2^ = 175.6; *P* < 0.001), as compared to mothers with at least one ITQOL outcome. The non-response analyses were repeated in all other ethnic subgroups separately and indicated the same pattern: non-responders were relatively more often lower educated, single parent and belonged to the younger age category than mothers with at least one ITQOL outcome. Mothers with missing data on ethnic background were more often lower educated (χ^2^ = 9.2; *P* = 0.01), single parents (χ^2^ = 12.2; *P* < 0.01), more often belonged to the younger age category (χ^2^ = 29.2; *P* < 0.01) and reported lower scores on some ITQOL scales (e.g., General Health Perceptions Mann–Whitney *U* = 273,868.5; *P* < 0.001), relative to mothers for whom ethnic background was known.

## Results

Characteristics of the study population are presented in Table 2. Significant differences between the ethnic subgroups were present in all variables, except for infant age, birth weight, gestational age, wheezing and the ITQOL respondent. Differences in maternal age (*F* = 72.8; *P* < 0.001), educational level (χ^2^ = 981.4; *P* < 0.001), family income (χ^2^ = 1,528.1; *P* < 0.001), marital status (χ^2^ = 568.2; *P* < 0.001), maternal psychopathology (*H*(7) = 701.3; *P* < 0.001) and parity (χ^2^ = 43.9; *P* < 0.001) were particularly great.

For seven out of nine ITQOL scales, infants from at least three ethnic minority groups presented significantly lower scores on health-related quality of life relative to infants classified as “Dutch” (*P* < 0.007; Table 3). Scores on the ITQOL scales were particularly lower in the following scales: Temperament and Moods, Family Activities and Family Cohesion. In the Temperament and Moods scale, the Turkish subgroup presented a median score of 70.8 (IQR, 15.3) relative to a median score of 80.6 (IQR, 13.9) in the Dutch subgroup (Mann–Whitney *U* = 269,204.5; *P* < 0.001). In general, infants from the European and Surinamese Creole subgroups presented more similar scores to the Dutch reference group than the other ethnic subgroups. This was particularly the case in the Physical Functioning scale (other European relative to Dutch: Mann–Whitney *U* = 533,273.5; *P* = 0.128*)* and the Growth and Development scale (Surinamese Creole relative to Dutch: Mann–Whitney *U* = 136,348.0; *P* = 0.549*)*. Infants from the Surinamese Creole subgroup were however the only subgroup that differed significantly from the Dutch subgroup on the Bodily Pain scale (Mann–Whitney *U* = 107,843.0; *P* < 0.001).

When stratifying by generational status, median scores of the first-generation group (*N* = 2,029) were significantly lower in all ITQOL scales, including the Bodily Pain scale, compared to native Dutch infants (*P* < 0.03). In the second-generation group (*N* = 847) median scores in a majority of the ITQOL scales were significantly lower compared to native Dutch infants (*P* < 0.03). Median scores in the Physical Functioning (*P* = 0.08) and Growth and Development (*P* = 0.51) scales did not differ significantly from the Dutch group.

Table 4 provides a series of hierarchical multivariate analyses illustrating the differences in infant health-related quality of life by maternal ethnic background, adjusted for confounders, infant health characteristics and family characteristics. After adjustment for confounders, a majority of the differences in ITQOL scale scores between the ethnic minority groups and the Dutch reference group remained significant (e.g., in the Temperament and Moods scale, children from the Turkish subgroup presented a score that was −10.7 % (−12.9; −8.2) lower than the Dutch reference group). After further adjustment for infant health characteristics (birth weight, gestational age, chronic conditions and wheezing), most differences remained significant; however, differences decreased significantly in the following scales: Bodily Pain (Turkish subgroup model 2 vs. model 1, *P* < 0.001), Temperament and Moods (Turkish subgroup model 2 vs. model 1, *P* < 0.001); General Health (Turkish group model 2 vs. model 1, *P* = 0.022); Parental Impact Emotional (Turkish subgroup model 2 vs. model 1, *P* < 0.001); and Family activities (Turkish subgroup model 2 vs. model 1, *P* = 0.016).

In model 3, the fully adjusted model additionally including family characteristics, further significant decreases on almost all ITQOL scales compared to model 2 were shown (e.g., Physical Functioning, Moroccan subgroup, *P* < 0.001). Most differences in infant health-related quality of life remained significant. However, the addition of family characteristics attenuated the differences to non-significance in the Physical Functioning scale (Surinamese Hindu, Moroccan and Turkish subgroups), the Growth and Development scale (Surinamese Hindu and Turkish subgroups), the General Health scale (Turkish subgroup), the Parental Impact Emotional scale (Antillean, Cape Verdean and Moroccan subgroups), the Parental Impact Time scale (Cape Verdean subgroup), the Family Activities scale (Surinamese Hindu and Turkish group) and the Family Cohesion scale (other European, Surinamese Creole, Surinamese Hindu and Moroccan and Turkish subgroups).

## Discussion

This large population-based cohort study showed that parent-reported health-related quality of life, even at the age of 12 months, is lower in infants from ethnic minority groups compared to native Dutch infants. Infant health characteristics (birth weight, gestational age at birth, presence of chronic conditions and presence of wheezing) and family risk factors (single parenthood, low educational level, family income and prenatal maternal psychopathology) mediated an important part of the association between the ethnic minority status and infant health-related quality of life. However, these factors could not fully explain all the ethnic differences in the ITQOL scale scores.

### Methodological considerations

This study is embedded in a longitudinal birth cohort. Parents and children are studied from early pregnancy onwards. Elaborate information on ethnic background, health-related quality of life, and child and family characteristics was available.

Several study limitations should be noted. In the study population, missing data were observed for maternal ethnic background and the ITQOL. The non-response analyses indicated that data on the ITQOL were more complete in children of higher educated, non-single and older mothers, a trend that was found in Dutch and non-Dutch infants. To check whether this selective attrition influenced our results, we estimated non-response probabilities and included these probabilities as weights in the comparisons of ITQOL scores (data not shown). Adjusting for this selective attrition did not substantially change the observed differences in infant health-related quality of life. Ethnic background was more often missing in participants who were younger and more often single, lower educated and those who reported lower health-related quality of life in their infants. It is possible that some of these mothers may have belonged to ethnic minority groups and that the differences that we found in terms of infant health-related quality of life may have been larger had this group been included. Our research assistants helped a few participants (all illiterate, mostly Berber and Moroccan mothers) with filling out the questionnaires. This may have influenced these participants’ report of infant health-related quality of life [28]. Maternal ethnic background was the main determinant in this study. This meant that paternal ethnic background was not considered. We checked whether results changed if paternal ethnic background was included instead of maternal ethnic background but did not observe a substantial change in results (data not shown).

The ITQOL is a parent-reported measure. In this study, we did not assess whether parents as proxies gave reliable ratings. We did adjust for relevant parent-related characteristics in the full model (single parenthood, low educational level, family income and maternal psychopathology) and found that some differences in infant health-related quality of life between ethnic minorities and the majority group remained present. Regardless, it is possible that the differences that we found may have been affected by parent-related characteristics other than the ones that we studied [29]. For instance, it may be possible that non-Dutch groups hold different beliefs about health and illness and may therefore report lower health-related quality of life scores in their infants [48, 49]. Studies have found that somatization, hypochondria and the expression of pain or discomfort have been found to vary by cultural background [50, 51]. Additionally, it is possible that, compared to Dutch groups, the threshold to report poor health-related quality of life is lower in non-Dutch groups.

### Health-related quality of life in infants from ethnic minority groups

Our study demonstrated that infants from most non-Dutch ethnic minority groups scored lower on almost all ITQOL scales. Ethnic differences were pronounced for Physical Functioning, Temperament and Moods, and the Family Cohesion scales. This confirms the results of studies among older child populations, adolescents and adults that also showed that minority groups are likely to score lower on various health indicators and measures of health-related quality of life [30–33].

Infants from the Surinamese Creole and European subgroups did not differ much from infants from the Dutch subgroup in terms of health-related quality of life. In fact, infants from the Surinamese Creole subgroup presented higher scores on the Bodily Pain scale than Dutch natives. Migration factors may explain why Surinamese Creole and European groups did not differ as much from the Dutch group, as they may have fewer difficulties adapting to the Dutch society due to the language and the longer migration history (Surinamese Creole) or culture (European) [34]. In the other ethnic minority groups that we studied, migration factors may have played a more prominent role. Firstly, acculturation factors (e.g., language barriers, discrimination) may lead to more stress. Studies show that stress during pregnancy exposes the fetus to elevated levels of stress hormones [35] possibly influencing fetal development; for example, maternal anxiety reduces the blood flow through the uterine arteries, which affects fetal development and possibly disease in later life [36]. This also explains why family risk factors and prenatal maternal psychopathology in particular explained an important part of the association between the ethnic minority status and infant health-related quality of life.

Additionally, acculturation factors have been found to influence access to and usage of health care services, including services for newborns and infants [30, 31, 37–41]. Lack of prompt medical care may lead to more health complications and possibly more worry about infant health [42]. Acculturation factors may be more important in first-generation ethnic minorities than in second-generation migrants and this may impact infant health-related quality of life [43]. In this study, we however found that both generational groups reported lower health-related quality of life in their infants compared to Dutch natives.

With regard to the parental impact scales, in our study, infants from ethnic minority groups also presented lower scores relative to those from the Dutch subgroup. On the one hand, this may reflect health issues in these children and their relatively low health-related quality of life scores. On the other hand, as noted by others [44, 45], the relatively low scores on parental impact scales may also be due to the strong family ties within ethnic minority groups relative to the “majority” group, and consequently, the infant’s health status may have a greater negative impact on the family as a whole.

It is noteworthy that the mediators that were included in our models were not always able to fully explain the relatively low infant health-related quality of life in the non-Dutch groups. Firstly, there may be additional maternal or infant health indicators and family characteristics not considered in this study that could explain the differences that we found. Secondly, it is conceivable that genetic factors play a role in explaining the ethnic differences in health-related quality of life in infants [46, 47].

## Conclusion

Parent-reported health-related quality of life, even at the age of 12 months, is lower in ethnic minority groups compared to native Dutch infants and could not be explained fully by infant health characteristics and family-related characteristics like single parenthood, low educational level and maternal psychopathology. We recommend further study to gain insight into the causes that underlie these differences. Firstly, it is important to gain more insight into genetic causes of differences in health-related quality of life among ethnic groups. Additionally, more research on cultural differences in perceptions of health-related quality of life is recommended. To gain more insight into the individual effects of migration and ethnicity, we recommend gathering reference data on infant health-related quality of life from the countries of origin of large migrant populations in Europe. In general, pediatricians should be aware of the ethnic inequalities in health-related quality of life and health status, even in early life. To decrease these inequalities, improving access to health care services for newborns and infants may be important. Additionally, programs aimed at reducing parental stress should be readily available to ethnic minority groups.

## Abbreviations

ITQOL: Infant Toddler Quality of Life
IQR: Inter-quartile range
SD: Standard deviation
PF: Physical functioning
GD: Growth and development
BP: Bodily pain
TM: Temperament and moods
GH: General health
PE: Parental impact emotional
PT: Parental impact time
FA: Family activities
FC: Family cohesion
